# Supplementary material for: Estradiol Removal by Adsorptive Coating of a Microfiltration Membrane
Source: Membranes (Basel). 2021 Jan 30;11(2):99. doi: 10.3390/membranes11020099 (PMC7911598; doi:10.3390/membranes11020099)
Supplement: Supplementary file 1 [file membranes-11-00099-s001.pdf]

membranes

Supplementary Material: Estradiol Removal by Adsorptive Coating on a Microfiltration Membrane

Zahra Niavarani, Daniel Breite, Andrea Prager, Bernd Abel and Agnes Schulze

Figure S1 shows the water contact angle test performed on modified membrane (PA-4) Values of five different (independently modified membranes) tests were averaged.

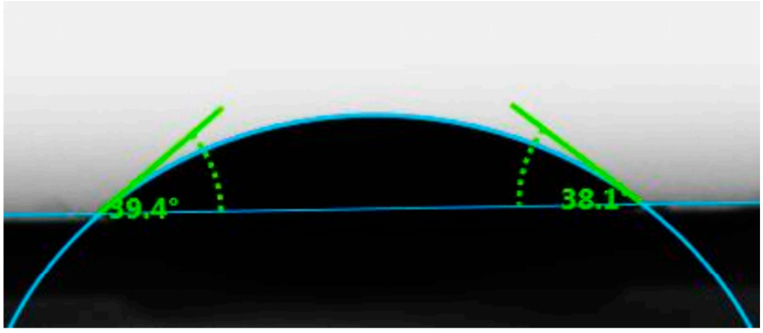

Figure S1. Water contact angle test on a modified membrane (PA-4).

Table S1 demonstrates the water contact angle values for the reference and modified membranes. Values of at least five different independently modified membranes were averaged.

Table S1. Values of Water contact angle for reference and modified polyethersulfone (PES) membrane.

	Water contact angle (°)	Error (°)
REF	43.95	3.82
PA[1]-1	42.26	4.12
PA-2	41.29	2.13
PA-3	43.49	4.4
PA-4	38.08	1.11
PA-5	39.52	4.95
PA-6	37.65	3.93
PA-7	38.35	3.56
PA-8	39.12	3.93
PA-9	40.89	3.32
PA-10	40.06	2.68
PA-11	40.21	3.28
PA-12	39.05	1.35
PA-13	40.12	2.42
PA-14	39.89	2.36
PA-15	38.18	3.13
PA-16	40.11	4.35

[1] Modified membranes (PA-1 to PA-16).

Table S2 presents the value of water permeance tests performed on reference and modified polyethersulfone (PES) membranes. Values of five different independently modified membranes are averaged.

Table S2. Values of water permeation tests of modified and reference PES membranes.

	**Water permeation ($mL \cdot min^{-1} \cdot cm^{-2} \cdot bar^{-1}$)**	**Error ($mL \cdot min^{-1} \cdot cm^{-2} \cdot bar^{-1}$)**
REF	40.1	2.3
PA-1	39.2	3.1
PA-2	39.8	2.5
PA-3	41	3.2
PA-4	41.3	3.4
PA-5	42.5	3.5
PA-6	40.3	2.9
PA-7	41.2	2.8
PA-8	40.5	2.7
PA-9	42.0	3.2
PA-10	40.1	3.5
PA-11	39.2	3.6
PA-12	41.3	3.4
PA-13	41.1	2.5
PA-14	40.8	2.9
PA-15	39.6	2.7
PA-16	39.3	2.7

Figure S2 illustrates the scanning electron microscope (SEM) images of modified and pristine polyethersulfone (PES) membranes of the top surface (top) and cross-section (bottom).

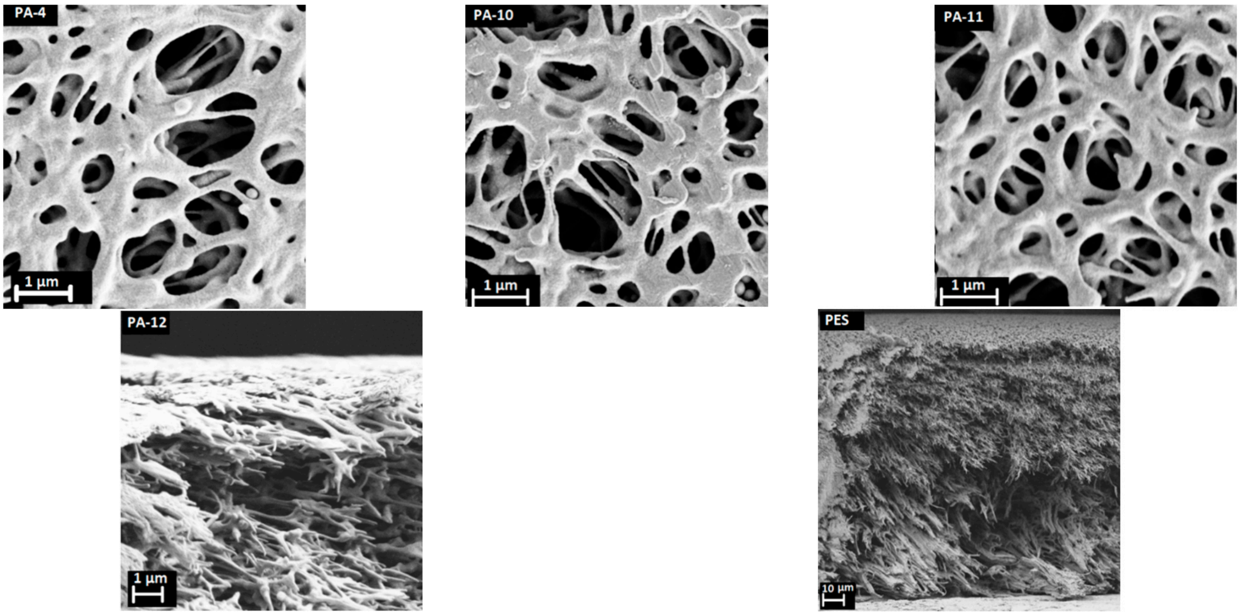

Figure S2. Scanning electron microscope (SEM) images of top surface (top) and cross-section (bottom) of modified and pristine polyethersulfone (PES) membrane at magnifications of 10,000 (Top) and 500 (bottom).

Table S3 demonstrates the adsorption (%) and adsorption capacity values for modified and reference PES membranes.

Table S3. Adsorption (%) and adsorption capacity (μg cm^{-2}) values for modified and pristine PES membrane.

	**E2 adsorption (%)**	**Adsorption capacity (μg cm^{-2})**
REF	35	0.44
PA-1	46	0.59
PA-2	60	0.76
PA-3	31	0.39
PA-4	43	0.55
PA-5	41	0.52
PA-6	64	0.82
PA-7	32	0.41
PA-8	43	0.55
PA-9	45	0.57
PA-10	64	0.82
PA-11	36	0.46
PA-12	52	0.66
PA-13	39	0.50
PA-14	48	0.61
PA-15	30	0.38
PA-16	51	0.65
